# Luteolin inhibits SH-SY5Y cell apoptosis through suppression of the nuclear transcription factor-κB, mitogen-activated protein kinase and protein kinase B pathways in lipopolysaccharide-stimulated cocultured BV2 cells

**DOI:** 10.3892/etm.2014.1564

**Published:** 2014-02-20

**Authors:** LIHONG ZHU, WEI BI, DAN LU, CHANJUAN ZHANG, XIAOMING SHU, DAXIANG LU

**Affiliations:** 1 Department of Pathophysiology, Institute of Brain Research, School of Medicine, Jinan University, Guangzhou, Guangdong 510632, P.R. China; 2 Department of Neurology, First Affiliated Hospital of Jinan University, Guangzhou, Guangdong 510630, P.R. China

**Keywords:** protein kinase B, luteolin, mitogen-activated protein kinase, microglia, neuroprotection, nuclear transcription factor κB, Toll-like receptor 4, neuroinflammation

## Abstract

Microglial activation is one of the causative factors for neuroinflammation, which is associated with the pathophysiology of neurodegenerative diseases. Our previous study showed that the flavonoid luteolin inhibited several pro-inflammatory enzymes and pro-inflammatory cytokines that are induced by activated microglia; however, its effect on signaling pathways is currently unknown. The present study examined the effects of luteolin on signaling pathways stimulated by lipopolysaccharide (LPS), including Toll-like receptor-4 (TLR-4), nuclear transcription factor-κB (NF-κB), mitogen-activated protein kinase (MAPK) family and protein kinase B (Akt) pathways in murine microglial BV2 cells. In addition, BV2 microglia and SH-SY5Y neuroblastoma cells were cocultured to observe the indirect neuroprotective effects of luteolin. Luteolin inhibited the LPS-stimulated expression of TLR-4. In addition, luteolin blocked LPS-induced NF-κB, p38, JNK and Akt activation, but had no effect on ERK. When SH-SY5Y cells were cocultured with LPS-stimulated BV2 microglia, pretreatment with luteolin increased neuronal viability and reduced the number of apoptotic cells. These data suggest that luteolin has a beneficial effect on neuroinflammatory events in neurodegenerative diseases via suppression of the NF-κB, MAPK and Akt pathways in activated microglial cells.

## Introduction

Microglia are the cells responsible for innate immunity in the central nervous system and accumulating evidence suggests that activated microglia modulate the development and/or progression of Alzheimer’s disease, Parkinson’s disease and amyotrophic lateral sclerosis, among other diseases (1–3). Exposure to lipopolysaccharide (LPS), β-amyloid or interferon (IFN)-γ activates microglia, inducing the secretion of a variety of pro-inflammatory mediators and potentially neurotoxic compounds (4,5). Receptor binding of cytokines stimulates a variety of intracellular signaling pathways that have been implicated in neurodegenerative disorders, including activation of nuclear transcription factor-κB (NF-κB), the mitogen-activated protein kinase (MAPK) family and protein kinase B (Akt). These are the most important molecules that control the synthesis and release of pro-inflammatory substances from activated microglia (6–8). Epidemiological studies suggest that inhibition of microglial activation attenuates the severity of neurotoxicity (9,10). The inhibition of activated microglia is therefore an important therapeutic target for neurodegenerative disorders.

Luteolin (3′,4′,5,7-tetrahydroxyflavone), a flavone that has been identified in celery, green pepper, perilla leaves and seeds, and at high concentrations in chamomile, exhibits strong anti-inflammatory, antioxidant and free-radical scavenging properties. In addition, luteolin has been shown to inhibit the LPS-induced production of tumor necrosis factor alpha (TNF-α) and nitric oxide (NO) in an activated macrophage-like cell line (11). Luteolin also reduces the production of LPS-induced pro-inflammatory cytokines in intestinal epithelial cells, mouse bone marrow-derived dendritic cells (12), rat fibroblasts (13) and human gingival fibroblasts (14). Furthermore, in a previous study we observed that luteolin inhibited the secretion of several pro-inflammatory enzymes and pro-inflammatory cytokines by activated microglia (15). However, the mechanism by which luteolin inhibits microglial inflammation is not completely understood. Much less is known about the role of luteolin in neuroprotection and regulation of the underlying signaling pathways.

In the present study, the effects of luteolin on Toll-like receptor-4 (TLR-4) expression and the NF-κB, MAPK and Akt signaling pathways were investigated using LPS-stimulated BV2 cells, a murine microglial cell line. In further experiments using a microglial-neuronal coculture system, the protective effects of luteolin against microglial-mediated LPS neurotoxicity, and therefore its potential role in the prevention of neurodegenerative diseases were investigated.

## Materials and methods

### Materials

Luteolin (purity > 98%; molecular weight, 286.24; chemical formula C_15_H_10_O_6_), LPS, dimethylsulfoxide (DMSO) and 3-(4,5-dimethylthiazol-2-yl)-2,5-diphenyltetrazolium bromide (MTT) were purchased from Sigma (St. Louis, MO, USA). Antibodies against NF-κB p65, p38, phosphorylated p38 (p-p38), JNK, phosphorylated JNK (p-JNK), ERK, phosphorylated ERK (p-ERK), Akt and phosphorylated Akt (p-Akt) were obtained from Cell Signaling Technology (Beverly, MA, USA). Antibodies against TLR-4 were obtained from Santa Cruz Biotechnology (Santa Cruz, CA, USA). Mouse anti-β-actin antibody was purchased from Sigma.

### Cell culture

BV2 immortalized murine microglia were provided by the Cell Culture Center of the Chinese Academy of Medical Sciences (Beijing, China). The human neuroblastoma cell line SH-SY5Y was donated by Dr Enxiang Tao. The cells were cultured in Dulbecco’s modified Eagle’s medium (DMEM) supplemented with 10% fetal bovine serum (FBS), 100 U/ml penicillin and 100 μg/ml streptomycin in a humidified atmosphere of 5% CO_2_ at 37°C. In all experiments, the BV2 microglia were pretreated with the indicated concentrations of luteolin for 1 h prior to the addition of LPS (1.0 μg/ml) in serum-free DMEM.

### Immunofluorescence staining

For immunofluorescence staining, the cells were fixed with 4% paraformaldehyde for 15 min, permeabilized with 0.1% Triton X-100 for 10 min and blocked with 5% bovine serum albumin (BSA) for 30 min. The cells were then incubated with primary antibody to NF-κB p65 (1:100 dilution) overnight at 4°C. After washing three times with PBS, the cells were incubated with secondary antibody conjugated to rhodamine for 1 h (Cell Signaling Technology). The nuclei were stained with Hoechst 33258. Fluorescent images were captured using a laser scanning confocal microscope (LSM 510 META; Carl Zeiss, Stuttgart, Germany).

### Total RNA isolation and quantitative PCR (qPCR) analysis

Total RNA was isolated with TRIzol reagent (Invitrogen Life Technologies, Carlsbad, CA, USA) according to the manufacturer’s instructions. Total RNA (1.0 μg) was reverse transcribed using M-MLV reverse transcriptase (Promega, Madison, WI, USA) to produce cDNA. The primers used for qPCR were as follows: *TLR-4*, 5′-GCT TTC ACC TCT GCC TTC AC-3′ and 5′-CCA ACG GCT CTG AAT AAA GTG-3′; and *GAPDH*, 5′-TCA CCA CCA TGG AGA AGG C-3′ and 5′-GCT AAG CAG TTG GTG GTG CA-3′. The following qPCR conditions were used: 40 cycles of denaturation at 94°C for 20 sec, annealing at 62°C for 30 sec and extension at 72°C for 30 sec. SYBR Green qPCR Master mix 2 (Takara Bio, Inc., Shiga, Japan) was used in all samples and the reactions were carried out in a 20-μl reaction volume using a LightCycler LC480 qPCR instrument (Roche Diagnostics, Basel, Switzerland). The mRNA expression levels of target genes relative to glyceraldehyde-3-phosphate dehydrogenase (*GAPDH*, a housekeeping gene used as an endogenous control) were calculated according to the standard curves.

### Western blot analysis

BV2 microglia were harvested and lysed in RIPA buffer [1 mM ethylenediaminetetraacetic acid (EDTA), 150 mM NaCl, 1% igepal (CA-630), 0.1% sodium dodecyl sulfate (SDS), 0.5 % sodium deoxycholate and 50 mM Tris HCl; pH 8.0 (Sigma)]. Equal amounts of protein were separated by 8–12% sodium dodecyl sulfate polyacrylamide gel electrophoresis (SDS-PAGE), transferred to polyvinylidene fluoride (PVDF) membranes, blocked with 5% nonfat milk for 2 h and incubated with primary antibodies at 4°C overnight. Following incubation with appropriate secondary antibodies conjugated to horseradish peroxidase (goat anti-rabbit secondary antibody was obtained from Cell Signaling Technology), immunoblots were exposed on film using electrochemiluminescence (ECL) western detection reagent (Amersham Pharmacia Biotech, Amersham, UK). The bands were quantified by the optical density ratio using β-actin as a control.

### Cytotoxicity assay in a coculture of microglia and neurons

SH-SY5Y cells were grown in the bottom of wells, BV2 cells were then grown in culture inserts (pore size 0.4 μm; Corning, New York, NY, USA) and 1.0 μg/ml LPS was added to the culture insert. In this coculture system, the microglia were able to communicate with the neurons through a semipermeable membrane, which avoids direct contact between the two cellular systems (16). After coculture for 24 h, the SH-SY5Y cells were incubated with MTT solution (0.5 mg/ml in PBS) for 4 h at 37°C. The culture supernatants were then removed, the resulting formazan crystals were dissolved in DMSO and the absorbance was read at 570 nm with a microplate reader (ReTiSoft Inc., Mannedorf, Switzerland). Cell survival was expressed as the ratio of absorbance (percentage survival) compared with a DMSO control.

### Detection of apoptosis in a coculture of microglia and neurons

In the coculture system described above, apoptotic SH-SY5Y cells were detected by the terminal deoxynucleotidyl transferase-mediated dUTP nick-end labeling (TUNEL) assay. Following each treatment, the TUNEL assay was performed according to manufacturer’s instructions (Roche Diagnostics Corporation, Indianapolis, IN, USA) and all nuclei were counterstained with 5 mg/ml Hoechst 33342 for 10 min at 37°C. The labeled SH-SY5Y cells were examined with a laser scanning confocal microscope. SH-SY5Y cells were considered to be apoptotic when their nuclei were costained with Hoechst 33342 and TUNEL. The number of apoptotic cells was counted among 100 randomly chosen neurons observed on several optic fields.

### Statistical analysis

Quantitative data are presented as the mean ± standard error of the mean (SEM) of at least three independent experiments. Comparisons between two groups were analyzed using the Student’s t-test. P<0.05 was considered to indicate a statistically significant result.

## Results

### Luteolin suppresses TLR-4 expression in LPS-stimulated BV2 microglia

**Figure 1:**
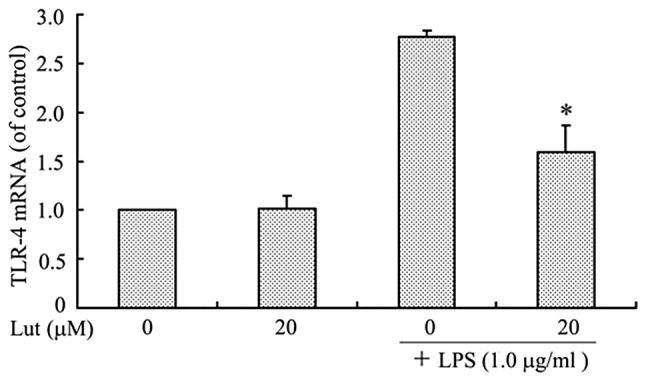
Effects of luteolin on the expression of TLR-4 in LPS-stimulated BV2 microglia. qPCR analysis of TLR-4 mRNA expression. Cells were treated with 20 μM luteolin for 1 h prior to the addition of LPS (1.0 μg/ml) for 1 h. Total RNA was then isolated and analyzed for TLR-4 mRNA expression using qPCR. TLR-4 mRNA expression levels were calculated relative to a GAPDH control using standard curves. Data were collected from three independent experiments each carried out in triplicate. ^*^Indicates a significant difference (P<0.05) relative to cells treated with LPS in the absence of luteolin. TLR-4, Toll-like receptor-4; LPS, lipopolysaccharide; GAPDH, glyceraldehyde-3-phosphate dehydrogenase; Lut, luteolin.

**Figure 2:**
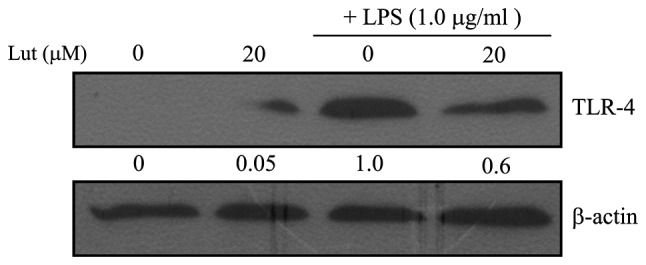
Effects of luteolin on the expression of TLR-4 in LPS-stimulated BV2 microglia. Western blot analysis of TLR-4. Cells were incubated with 20 μM luteolin for 1 h prior to incubation with LPS (1.0 μg/ml) for 24 h. Cell lysates were then prepared and subjected to western blotting. TLR-4 protein expression was quantified by OD ratio using β-actin as a control. Data were collected from three independent experiments each carried out in triplicate. ^*^Indicates a significant difference (P<0.05) relative to cells treated with LPS in the absence of luteolin. TLR-4, Toll-like receptor-4; LPS, lipopolysaccharide; OD, optical density; Lut, luteolin.

To examine the effect of luteolin on TLR-4 expression, the levels of TLR-4 mRNA and protein in LPS-stimulated BV2 microglia were measured. The BV2 microglia were pretreated with luteolin for 1 h and then stimulated with LPS for 1 h prior to qPCR or for 24 h prior to western blotting. As shown in Figs. 1 and 2, TLR-4 mRNA and protein levels increased in LPS-stimulated BV2 microglia, but both were markedly suppressed by treatment with luteolin. This result indicates that luteolin suppressed TLR-4 expression and therefore may lead to inhibition of activation of the NF-κB, MAPK and Akt pathways.

### Effects of luteolin on the NF-κB signaling pathway

**Figure 3:**
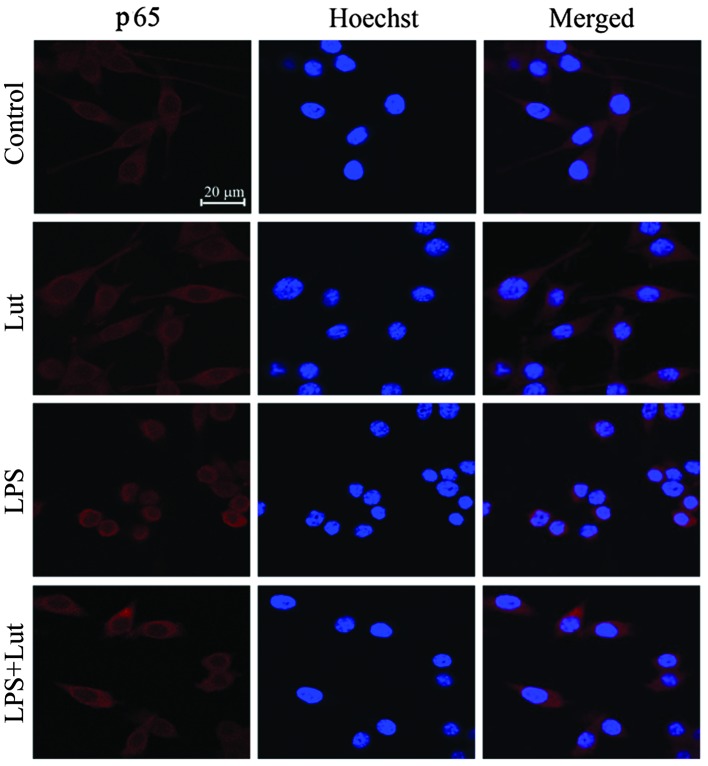
Downregulation of NF-κB activation by luteolin in LPS-stimulated BV2 microglia. Immunofluorescent staining showing the cellular distribution of the NF-κB p65 subunit (red). Cells were pretreated with luteolin for 1 h, followed by LPS (1.0 μg/ml) stimulation for 1 h. Hoechst 33258 (blue) was used to visualize the nuclei. Data was collected from three independent experiments each carried out in triplicate. Scale bar represents 20 μm. LPS, lipopolysaccharide; NK-κB, nuclear transcription factor κB; Lut, luteolin.

Activation of NF-κB leads to its translocation to the nucleus where it mediates the transcriptional regulation of pro-inflammatory genes. The activation and nuclear translation of NF-κB is a key step in LPS-stimulated microglial activation. The regulation of NF-κB by luteolin was investigated using immunofluorescence staining. As shown in Fig. 3, the NF-κB p65 subunit was primarily retained in the cytoplasm in unstimulated cells; however, following stimulation with LPS, cytoplasmic NF-κB p65 levels were reduced, with a corresponding increase in nuclear NF-κB p65. Treatment with 20 μM luteolin significantly blocked the activation of NF-κB p65 nuclear translocation in LPS-stimulated BV-2 cells. This result suggests that luteolin suppresses pro-inflammatory enzymes and pro-inflammatory cytokines by inhibiting NF-κB activation.

### Effects of luteolin on the MAPK signaling pathway

**Figure 4:**
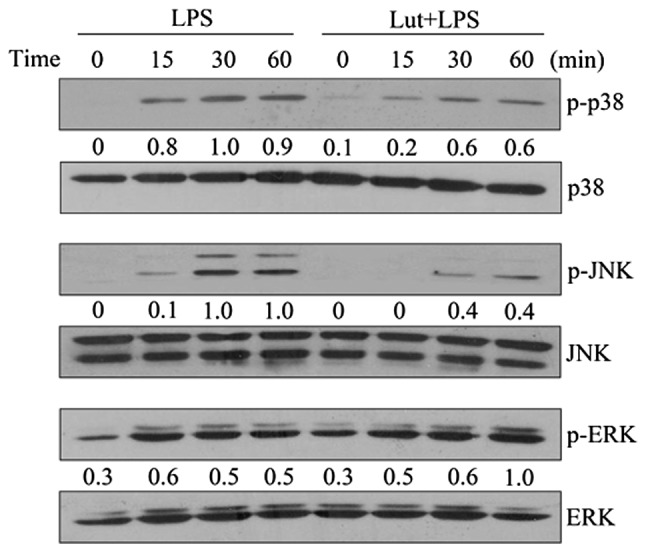
Downregulation of MAPK phosphorylation by luteolin in LPS-stimulated BV2 microglia. BV2 microglial cells were treated with LPS (1.0 μg/ml) for the indicated time with or without pretreatment with 20 μM luteolin for 1 h. Cell lysates were prepared and used for western blot analysis with antibodies against the indicated MAPKs. Expression levels of phosphorylated MAPKs were quantified by OD ratios relative to controls, and these data are shown between the relevant blots. LPS rapidly activated MAPKs including p38, JNK, and ERK within 15 min of LPS stimulation, while pretreatment of cells with 20 μM luteolin markedly inhibited the LPS-induced phosphorylation of p38 and JNK. Data were collected from three independent experiments each carried out in triplicate. MAPK, mitogen-activated protein kinase; LPS, lipopolysaccharide; OD, optical density.

To investigate whether the inhibition of NF-κB activation by luteolin is mediated via the MAPK pathway, the phosphorylation of three MAPK molecules, p38 MAPK, JNK and ERK1/2, was examined in LPS-stimulated BV-2 cells. As shown in Fig. 4, LPS rapidly activated MAPKs within 15 min of LPS stimulation, while luteolin at 20 μM markedly inhibited the LPS-induced phosphorylation of p38 and JNK, but had no effect on ERK phosphorylation. The levels of non-phosphorylated p38, JNK and ERK were unaffected by LPS or luteolin treatment.

### Effects of luteolin on the Akt signaling pathway

**Figure 5:**
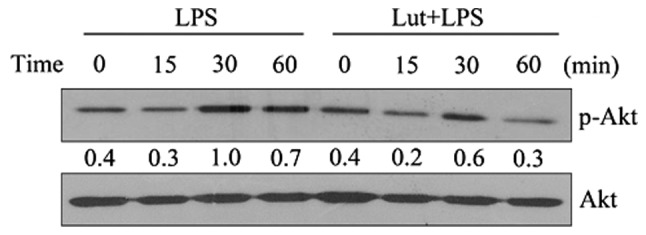
Downregulation of Akt phosphorylation by luteolin in LPS-stimulated BV2 microglia. The BV2 microglial cells were treated with LPS (1.0 μg/ml) for the indicated time with or without pretreatment with 20 μM luteolin for 1 h. Cell lysates were prepared and used for western blot analysis with antibodies against Akt and p-Akt. Expression levels of p-Akt were quantified by OD ratios relative to control. LPS treatment induced Akt phosphorylation, this process was significantly suppressed by luteolin. Data were collected from three independent experiments each carried out in triplicate. Akt, protein kinase B; LPS, lipopolysaccharide; p-Akt, phosphorylated protein kinase B; Lut, luteolin; OD, optical density.

The effect of luteolin on Akt was then examined. As shown in Fig. 5, luteolin significantly inhibited the LPS-induced activation of Akt. The results suggest that inhibition of Akt by luteolin may contribute to the suppression of LPS-induced NF-κB activation and the expression of inflammatory mediators in BV2 cells.

### Luteolin decreases microglial-induced SH-SY5Y cell death in a coculture system

**Figure 6:**
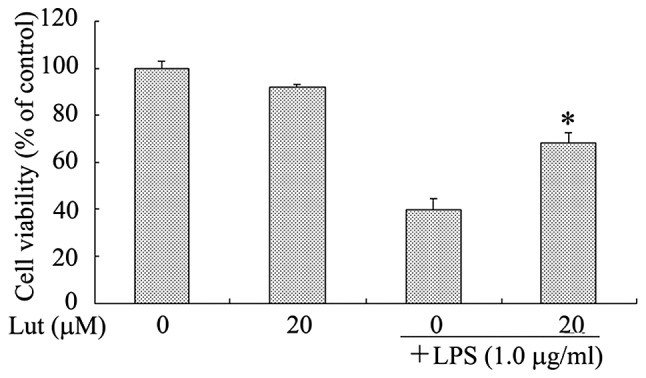
Effect of luteolin on SH-SY5Y cell survival in an LPS-induced microglial-neuronal coculture system. SH-SY5Y cells were cocultured with LPS-activated BV2 microglia with or without pretreatment with 20 μM luteolin for 24 h. An MTT assay was used to determine SH-SY5Y cell viability. ^*^P<0.05 compared with the LPS group. Lut, luteolin; LPS, lipopolysaccharide; MTT, 3-(4,5-dimethylthiazol-2-yl)-2,5-diphenyltetrazolium bromide.

In order to investigate whether luteolin protects against the neuronal death induced by microglial activation, a coculture system with SH-SY5Y neuronal cells and BV2 microglia was used. LPS at concentrations of 0.1, 1.0 or 10.0 μg/ml were not observed to induce cell death in the SH-SY5Y cells (data not shown). Next, the SH-SY5Y cell viability following coculture with LPS-activated BV2 microglia was examined using the MTT assay. As shown in Fig. 6, SH-SY5Y cells in control inserts in the absence of LPS-stimulated BV2 microglia did not undergo cell death. By contrast, LPS treatment alone led to a high level of SH-SY5Y cell death in the coculture, suggesting that the LPS-activated microglia secreted pro-inflammatory cytokines that were able to migrate through the insert, inducing the death of the neuronal cells. Treatment with luteolin markedly reduced the death of the SH-SY5Y cells; cell viability was increased by ~28.6% when the LPS-stimulated BV2 microglia were pretreated with luteolin (Fig. 6).

### Apoptosis was determined by the TUNEL assay

**Figure 7:**
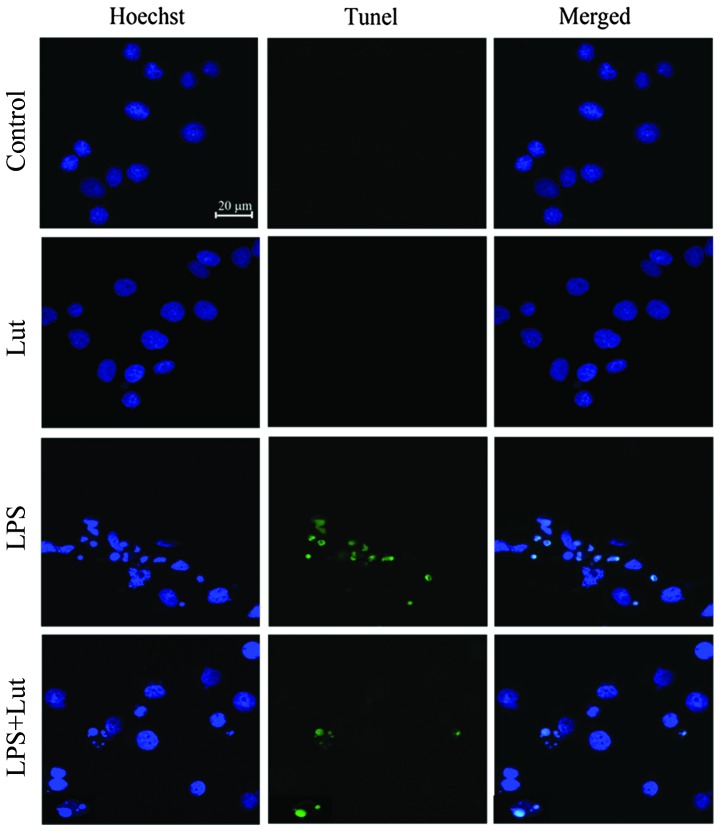
Effect of luteolin on SH-SY5Y cell survival in an LPS-induced microglial-neuronal coculture system. SH-SY5Y cells were cocultured with LPS-activated BV2 microglia with or without pretreatment with 20 μM luteolin for 24 h. Immunofluorescence detection of apoptotic SH-SY5Y cells cocultured with BV2 microglia. Scale bar represents 20 μm. Lut, luteolin; LPS, lipopolysaccharide.

**Figure 8:**
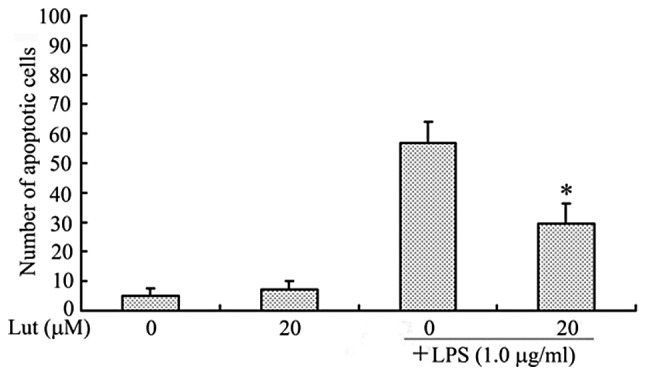
Effect of luteolin on SH-SY5Y cell survival in an LPS-induced microglial-neuronal coculture system. SH-SY5Y cells were cocultured with LPS-activated BV2 microglia with or without pretreatment with 20 μM luteolin for 24 h. The number of apoptotic neurons counted on double-stained slides (merged) from a total of 100 nuclei. Data were collected from three independent experiments each carried out in triplicate. ^*^Indicates a significant difference (P<0.05) relative to cells treated with LPS in the absence of luteolin. Lut, luteolin; LPS, lipopolysaccharide.

As shown in Fig. 7, SH-SY5Y nuclei were stained with Hoechst 33342 (blue) and apoptotic neurons were stained using the TUNEL technique (green). Coculture with BV2 microglia exposed to LPS alone resulted in a significant increase in the number of apoptotic SH-SY5Y cells compared with the number of untreated cells. The administration of luteolin reduced the number of apoptotic SH-SY5Y cells (Fig. 8). These results clearly demonstrate that luteolin protected neurons from microglial-mediated LPS neurotoxicity, supporting its potential role in the prevention of neurodegenerative diseases.

## Discussion

The flavonoid luteolin has been shown to inhibit LPS-induced IL-6 production in the brain by inhibiting the JNK signaling pathway and the activation of AP-1 in microglia (17). Luteolin also suppressed microglial TNF-α and IL-6 production stimulated by IFN-γ in the presence of CD40 ligation, and markedly inhibited the IFN-γ-induced phosphorylation of STAT1 (18). These data suggest that this flavonoid is a potent modulator of microglial activation and affects several signaling pathways, leading to a unique phenotype with anti-inflammatory, anti-oxidative and neuroprotective characteristics (19). Previous findings suggest dietary luteolin enhances spatial working memory by mitigating microglial-associated inflammation in the hippocampus (20). Our previous observations confirm the inhibitory effects of luteolin on pro-inflammatory cytokine expression in microglia (15). However, the mechanism by which luteolin mediates these anti-inflammatory effects on microglia is not completely understood and few studies have explored the impact of luteolin on neuroprotection.

*In vitro*, microglia may be activated experimentally with the bacterial cell wall component LPS. Internalization of TLR-4, rendering microglial cells less sensitive to activation by LPS, plays a role in inducing a reduction in TNF-α production by BV-2 microglial cells (21). TLR-4 is a member of the TLR family of pattern recognition receptors that generate innate immune responses to pathogens by activating a cascade of pro-inflammatory events (22). Therefore, treatments that attenuate TLR-4-associated inflammatory cascades may prove beneficial in ameliorating microglial activation and preventing neurodegenerative processes. The results of the present study indicate that luteolin pretreatment inhibited the upregulation of TLR-4 expression induced by LPS at the transcriptional and translational levels. It was therefore hypothesized that the underlying molecular mechanisms involved include interference with the LPS-triggered increase in TLR-4 expression. The results of the present study indicate that luteolin may inhibit NF-κB, p38, JNK, MAPK and Akt activation through the suppression of TLR-4 expression.

Activation of NF-κB leads to its translocation to the nucleus where it mediates the transcriptional regulation of pro-inflammatory genes (23). The activation and nuclear translation of NF-κB is a key step in LPS-stimulated microglial activation (24). In the current study, treatment with 20 μM luteolin significantly blocked NF-κB p65 nuclear translocation in LPS-stimulated BV-2 cells. In our previous study, a luciferase reporter assay was performed to investigate the possibility that luteolin inhibits NF-κB transcriptional activity, and the possibility that luteolin blocks the phosphorylation and subsequent degradation of IκB in LPS-induced BV2 cells was investigated; it was observed that luteolin causes a marked inhibition of NF-κB p65 nuclear translocation (15). These results, also confirmed by the current study, suggest that luteolin suppresses pro-inflammatory enzymes and pro-inflammatory cytokines through the inhibition of NF-κB activation.

There is evidence that MAPKs play a key role in the regulation of the synthesis and release of pro-inflammatory substances by activated microglia (25). LPS is known to activate various MAPKs, including p38, JNK and ERK. The MAPKs tested in this study (p38, JNK and ERK1/2) were activated in glia and neurons following LPS treatment, suggesting their involvement in glial activation and the neuronal response to diffusible, glia-derived neurotoxic molecules (26). To investigate whether the inhibition of NF-κB activation by luteolin is mediated via the MAPK pathway, the phosphorylation of three MAPK molecules, p38 MAPK, JNK and ERK1/2 in LPS-stimulated BV-2 cells was examined. LPS rapidly activated MAPKs within 15 min of LPS stimulation, while luteolin at 20 μM markedly inhibited the LPS-induced phosphorylation of p38 and JNK, but had no effect on ERK. However, further studies are necessary to support this conclusion.

Multiple signaling pathways, such as those involving MAPKs and Akt, are involved in LPS-stimulated signal transduction and lead to the activation of NF-κB and the subsequent induction of pro-inflammatory gene expression (27,28). Akt is activated via the phosphoinositide-3-OH kinase (PI3K) pathway, an important pathway regulating inflammation and immunity (29). The results of the present study indicate that luteolin also significantly inhibited the LPS-induced activation of Akt. The data suggest that inhibition of Akt by luteolin may contribute to the suppression of LPS-induced NF-κB activation and the expression of inflammatory mediators in BV2 cells.

Neurotoxic microglial-neuronal interactions have been implicated in the pathogenesis of various neurodegenerative diseases (30). Microglial activation has been shown to promote the production of inflammatory cytokines leading to neuronal apoptosis (31,32). In order to investigate whether luteolin is able to rescue neurons from death induced by microglial activation, SH-SY5Y cells and BV2 microglia in a coculture system were used in the present study. The results clearly indicate that when SH-SY5Y cells were cocultured with LPS-stimulated BV2 microglia, neuronal cell death increased by 60.2% and the number of apoptotic neurons increased by 57.0%. However, treatment with luteolin in this LPS-induced coculture system increased cell viability by 28.6% and reduced the apoptotic cell number by 27.0%. These data suggest that luteolin inhibited SH-SY5Y cell apoptosis via inhibition of microglial activation in the microglial-neuronal coculture system. These results provide strong evidence that luteolin protects neurons from microglial-mediated LPS neurotoxicity. However, further *in vivo* investigation of this activity is necessary in order to clarify the molecular mechanisms involved and assess the full medicinal potential of luteolin.

In conclusion, the present study demonstrated that luteolin inhibited the LPS-stimulated expression of TLR-4. Luteolin also blocked LPS-induced NF-κB, p38, JNK and Akt activation, but had no effect on ERK. When SH-SY5Y cells were cocultured with LPS-stimulated BV2 microglia, pretreatment with luteolin increased neuronal viability and reduced the number of apoptotic cells. These observations suggest that luteolin has a therapeutic application in the treatment of neurodegenerative diseases.
